# Supplementary material for: Aqueous Binary Mixtures of Stearic Acid and Its Hydroxylated Counterpart 12-Hydroxystearic Acid: Fine Tuning of the Lamellar/Micelle Threshold Temperature Transition and of the Micelle Shape
Source: Molecules. 2023 Aug 29;28(17):6317. doi: 10.3390/molecules28176317 (PMC10489131; doi:10.3390/molecules28176317)
Supplement: Supplementary file 1 [file molecules-28-06317-s001.zip › molecules-2554662-supplementary.pdf]

# Aqueous Binary Mixtures of Stearic Acid and Its Hydroxylated Counterpart 12-Hydroxystearic Acid: Fine Tuning of the Lamellar/Micelle Threshold Temperature Transition and of the Micelle Shape

**Maëva Almeida [1,2], Daniel Dudzinski [2], Bastien Rousseau [1], Catherine Amiel [1], Sylvain Prévost [3], Fabrice Cousin [2,*] and Clémence Le Coeur [1,2,*]**

[1] CNRS, ICMPE, UMR 7182, 2 Rue Henri Dunant, Université Paris Est Creteil, 94320 Thiais, France; maeva.ferreira-almeida@u-pec.fr (M.A.); bastien.rousseau@etu.u-pec.fr (B.R.); catherine.amiel-guenoun@cnrs.fr (C.A.)

[2] Laboratoire Léon Brillouin, Université Paris-Saclay, CEA-CNRS UMR 12 CEA Saclay, 91191 Gif sur Yvette, France; daniel.dudzinski@cea.fr

[3] Institut Laue-Langevin, 71 Avenue des Martyrs, CS 20156, CEDEX 9, 38042 Grenoble, France; prevost@ill.fr

* Correspondence: fabrice.cousin@cea.fr (F.C.); clemence.le-coeur@cnrs.fr (C.L.C.)

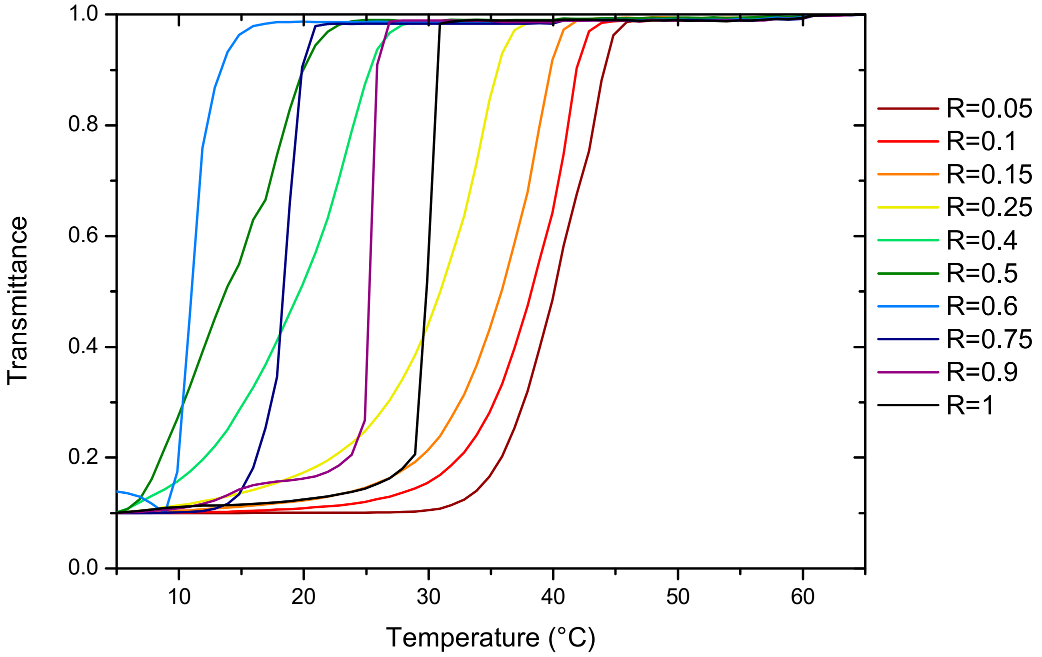


**Figure S1.** Transmittance as a function of temperature for HSA/SA mixtures from R=0 to R=1 upon cooling at a cooling rate of 1°C/min

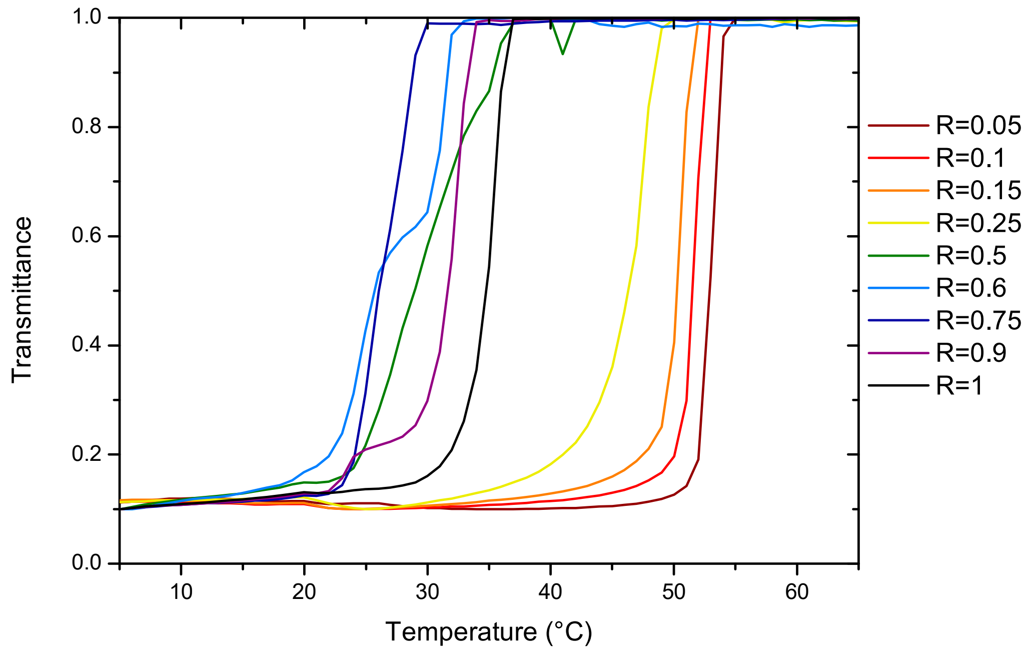


**Figure S2.** Transmittance as a function of temperature for HSA/SA mixtures from R=0 to R=1 upon heating at a heating rate of 0.2°C/min

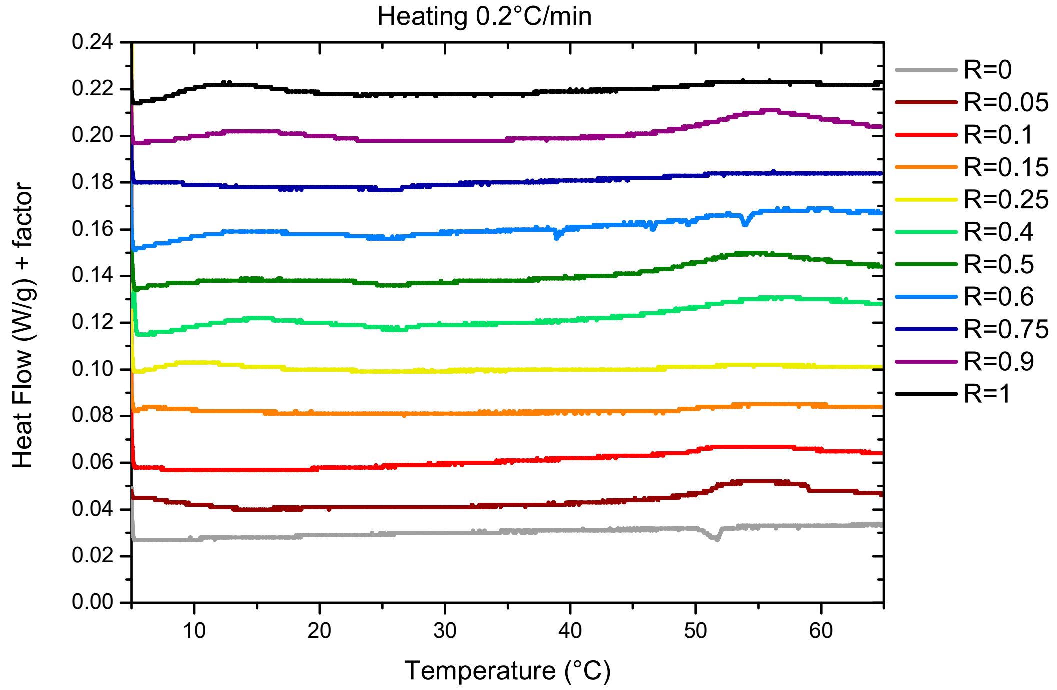


**Figure S3.** Enthalpograms obtained for 2wt% mixture in fatty acid at various R ratios upon heating at a heating rate of 0.2°C/min. Data are shifted for clarity.

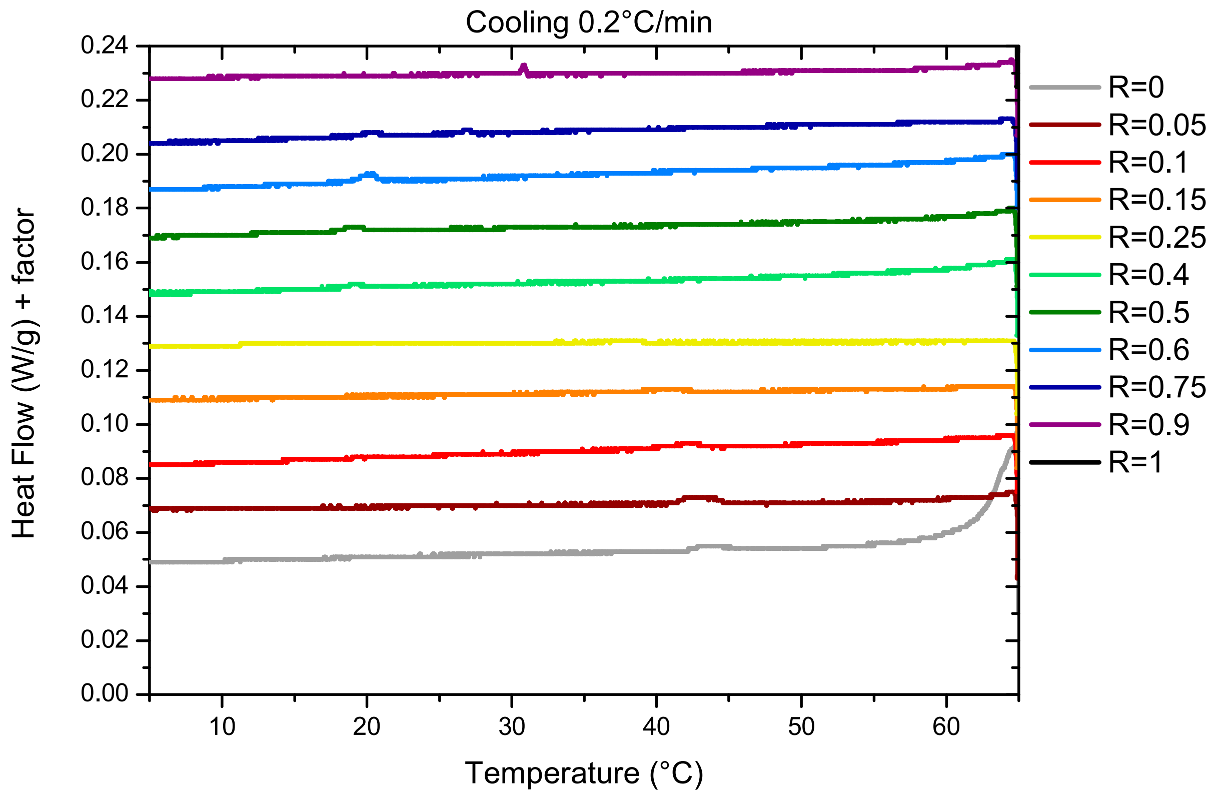


**Figure S4.** Enthalpograms obtained for 2wt% mixture in fatty acid at various R ratios upon cooling at a cooling rate of 0.2°C/min. Data are shifted for clarity.

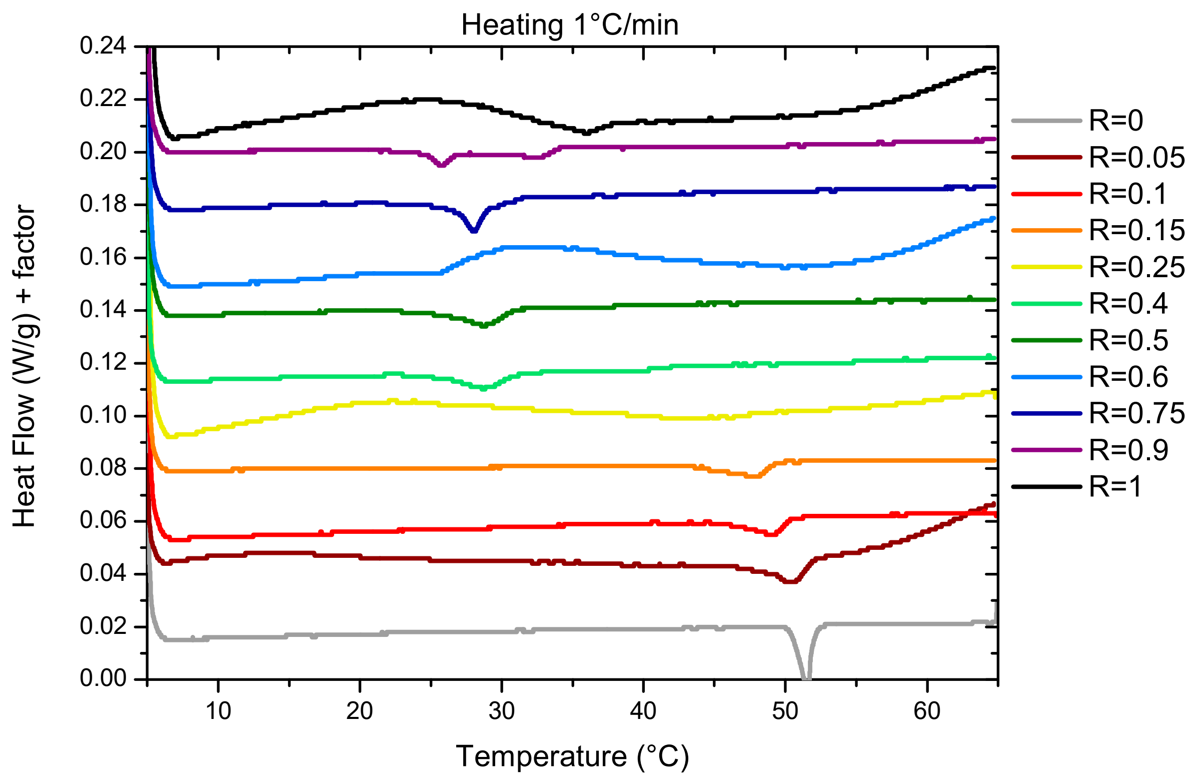


**Figure S5.** Enthalpograms obtained for 2wt% mixture in fatty acid at various R ratios upon heating at a heating rate of 1°C/min. Data are shifted for clarity.

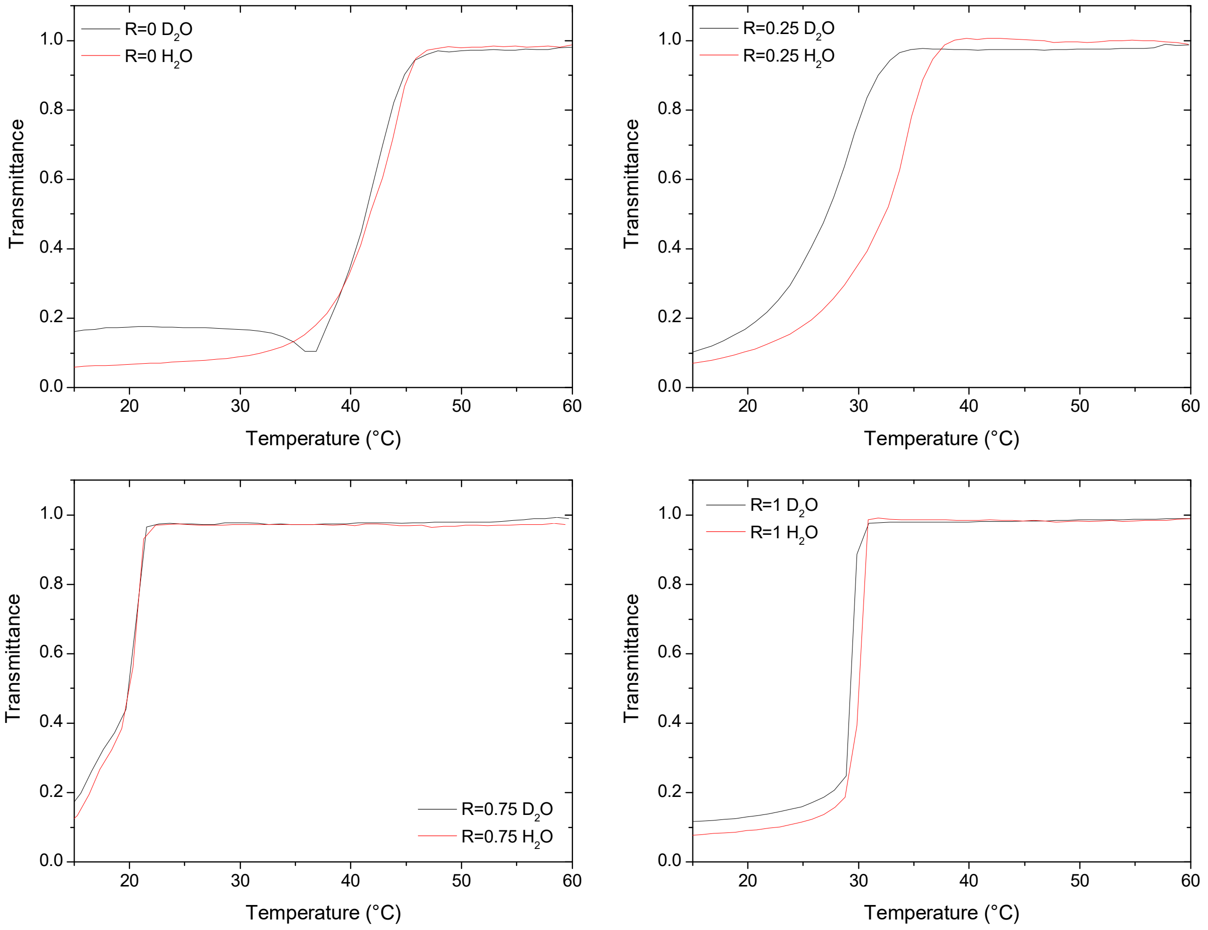


**Figure S6.** Comparison of transmittance of R=0, R=0.25, R=0.75 and R=1 samples in $D_2O$ and in $H_2O$.

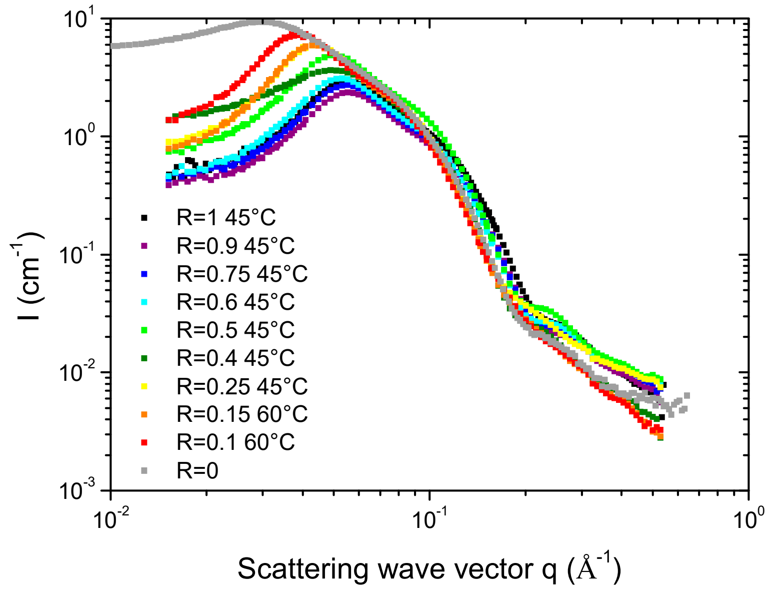


**Figure S7.** SANS intensity profiles at 45°C for the samples in $D_2O$ with R ratios between 0.25 and 1, and 60°C for the samples with ratio between 0 and 0.15.

**SANS data fitting**

All spectra were fitted with an ellipsoid model present in SasView 5.0.4 (http://www.sasview.org/.). The form factor of an ellipsoid object is given by $P(q, \alpha) = \frac{scale}{V} F^2(q, \alpha) + background$.[1] The structure factor, S(q), which quantifies the intermicellar interactions, was fitted with an "Hayter_MSA" model (Hayter and Penfold model) displayed in the SASview software. This accounts for a repulsive screened coulombic intermicellar interaction potential. We fixed the scattering length density of the solvent and the fatty acids, the volume fraction, the salt concentration, the dielectric constant and the temperature. The others parameters were fitted and are present in the Table 1.

| Ratio | Polar Radius (Å) | Equatorial Radius (Å) | Charge (e-) |
|---|---|---|---|
| **0** | 18.8 ± 0.2 | 38.8 ± 0.9 | 15 |
| **0.1** | 21.0 ± 0.3 | 32.0 ± 0.2 | 42 |
| **0.15** | 22.3 ± 0.3 | 28.0 ± 0.6 | 26 |
| **0.25** | 24.9 ± 0.2 | 25.2 ± 0.3 | 44 |
| **0.5** | 25.9 ± 0.3 | 21.8 ± 0.4 | 32 |
| **0.6** | 26.0 ± 0.4 | 20.0 ± 0.3 | 27 |
| **0.75** | 28.4 ± 0.8 | 18.7 ± 0.4 | 40 |
| **0.9** | 31.0 ± 0.5 | 18.5 ± 0.5 | 46 |
| **1** | 32.0 ± 0.8 | 18.5 ± 0.4 | 32 |

**Table S1.** Results obtained by fitting the micelles by elliptical sphere at 45°C or 60°C depending of the ratio R

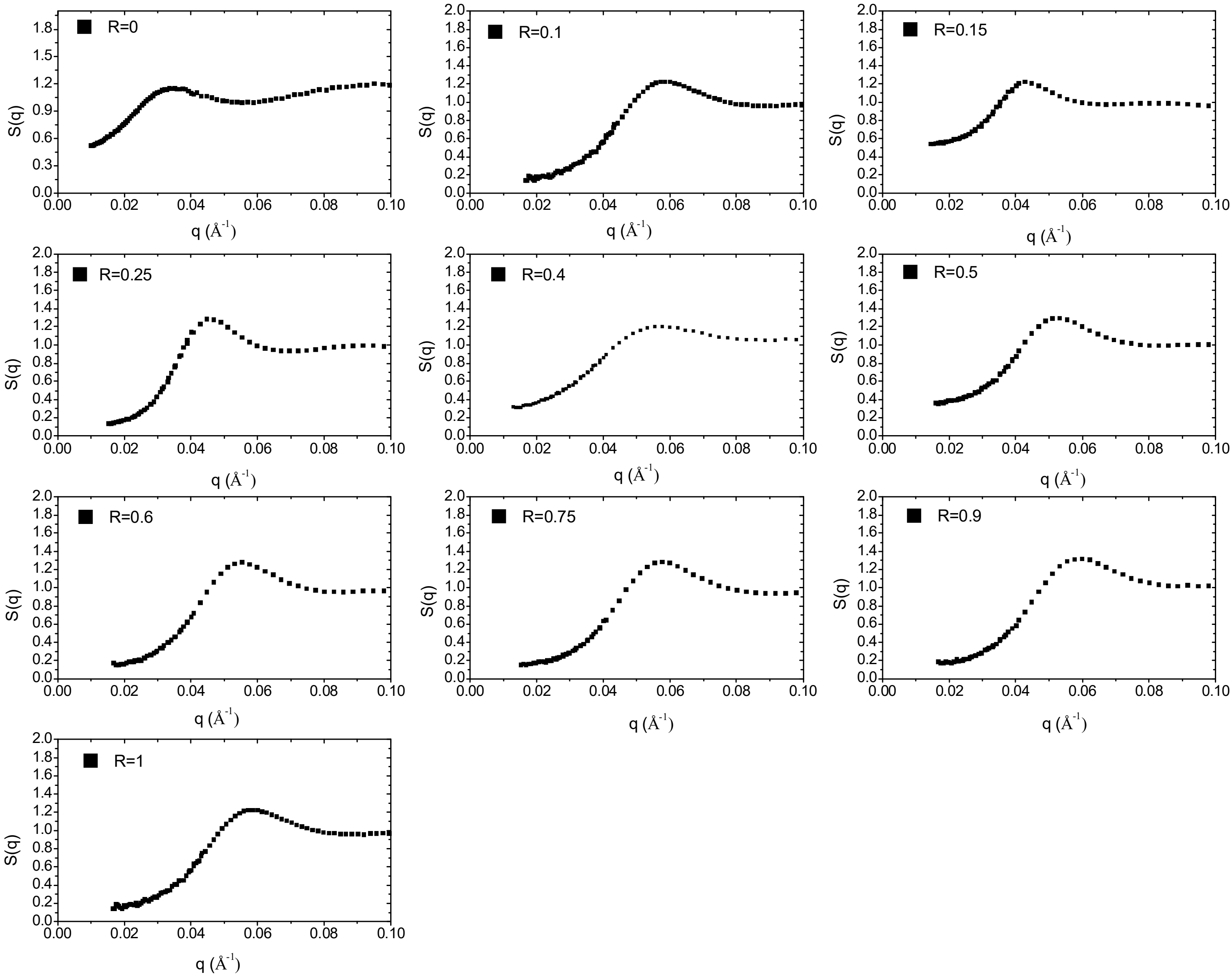


**Figure S8.** Structure factors of micelles obtained by SANS at 45° or 60°C depending on the ratio R.

**References**

(1) L. A. Feigin and D. I. Svergun. Structure Analysis by Small-Angle X-Ray and Neutron Scattering, Plenum Press, New York, 1987
